# A Novel Designed Bioreactor for Recovering Precious Metals from Waste Printed Circuit Boards

**DOI:** 10.1038/srep13481

**Published:** 2015-08-28

**Authors:** Ruan Jujun, Zheng Jie, Hu Jian, Jianwen Zhang

**Affiliations:** 1 School of Environmental Science and Engineering, Sun Yat-Sen University, Guangzhou, People’s Republic of China; 2 School of Environmental Science and Engineering, Yangzhou University, Yangzhou, People’s Republic of China

## Abstract

For recovering precious metals from waste printed circuit boards (PCBs), a novel hybrid technology including physical and biological methods was developed. It consisted of crushing, corona-electrostatic separation, and bioleaching. Bioleaching process is the focus of this paper. A novel bioreactor for bioleaching was designed. Bioleaching was carried out using *Pseudomonas chlororaphis.* Bioleaching experiments using mixed particles of Au and Cu were performed and leachate contained 0.006 mg/L, 2823 mg/L Au^+^ and Cu^2+^ respectively. It showed when Cu existed, the concentrations of Au were extremely small. This provided the feasibility to separate Cu from Au. The method of orthogonal experimental design was employed in the simulation bioleaching experiments. Experimental results showed the optimized parameters for separating Cu from Au particles were pH 7.0, temperature 22.5 °C, and rotation speed 80 r/min. Based on the optimized parameters obtained, the bioreactor was operated for recovering mixed Au and Cu particles. 88.1 wt.% of Cu and 76.6 wt.% of Au were recovered. The paper contributed important information to recover precious metals from waste PCBs.

Abundant electronic waste (e-waste) has been produced following the dramatically increasing of electronics products. The global production of e-waste is about 20–25 million tons per year, while China produced about 2.5 million tons^1^. As the core component of electronics products, big amount of waste printed circuit boards (PCBs) are generated^2^.

PCB contains nearly 28% metals and the purity of metals in PCB is greater than that of rich-content minerals. Many small backyards workshops adopt acid-washing and open incineration to recover metals from waste PCBs. Serious pollutions have been caused by the methods of acid-washing and open incineration such as emissions of polybrominated diphenyl ethers (PBDEs), polychlorinated dibenzo-*p*dioxins and dibenzofurans (PCDD/Fs)^3^, and heavy metals^4^,^5^. Thus, environment-friendly and effective technologies are the pressing demand of treating waste PCBs.

Physical methods have been the preferred technologies for treating e-waste^6^,^7^, especially for waste PCBs^8^. An environmental friendly automatic line for recovering metals from waste PCBs was developed in previous work^9^. The line can recover 95% metals of PCBs by corona-electrostatic separation. However, many precious metals are contained in waste PCBs^10^. For instance, PCB of mobile phone contained about 0.0043% Au and 0.054% Ag^11^. In corona-electrostatic separation, precious metals (Au or Ag), present in waste PCBs in trace concentrations, were missed. In fact, physical treatment presents inability to recover precious metals. The reasons are: (1) precious metals take up a small proportion in waste PCBs. Developing exclusive mechanical devices to recover precious metals will result in high cost. The cost of mechanical devices cannot be offset by the bits of recovered precious metals; (2) the small mass and inert nature of precious metals bring difficulties to separate them by ordinary physical separation. Thus, how to recover precious metals from waste PCBs is significant for improving additional value of e-waste disposal.

Bioleaching is widely used in mineral processing^12^,^13^. It is an environment-friendly and low-cost method. Currently, application of bioleaching for recovering metals from waste PCBs was reported^14^. For recovering precious metals from waste PCBs, cyanogenic strains were reported to be useful in the recovery of Au and Ag from waste PCBs. *Chromobacterium violaceum* and *Pseudomonas aeruginosa* were the most reported strains^15^,^16^. However, this application is still in its infancy. The difficulty is how to separate nonmetallic parts when using bioleaching method for recovering metals from waste PCBs in industrial production. Nonmetals of waste PCBs will bring negative effects to microbe and deteriorate the leaching process. Thus, it is difficult to recover precious metals from crushed waste PCBs if just bioleaching was employed. Meanwhile, the published cyanogenic strains were not suitable to be employed in industrial application. *Chromobacterium violaceum* was pathogenic bacteria and *Pseudomonas aeruginosa* existed in sputum. Additionally, little information is available about the reactor which was used for bioleaching precious metals from crushed waste PCBs^17^.

In this study, a novel hybrid technology of corona-electrostatic separation and bioleaching was proposed to recover Au from waste PCBs. Corona-electrostatic separation was used to separate nonmetals parts of crushed waste PCBs. Bioleaching was employed to recover precious metals from crushed waste PCBs. A novel bioreactor was designed for bioleaching. Bioleaching experiments of separating Ag and Cu particles were performed. This technology can recover Au from waste PCBs and contribute to the aspect of high-value recycling of e-waste.

## Design parts

### Design of the hybrid technology for recovering precious metals in waste PCBs

In the environmental friendly line for recovering metals from waste PCBs, waste PCB was crushed to particles (size of 0.6 mm) for liberating the materials. Then, Metallic and nonmetallic materials were separated by corona-electrostatic separation. However, it was hard to separate each metal by corona-electrostatic separation. Mixed metallic particles were obtained and they were sold as Cu. The precious metals (Au and Ag) were lost. Thus, how to recover the lost precious metals is needed to be concern. The main technologies of separating metals of waste PCBs were compared in follows.

*Chemical leaching*: this technology had high separation rate of metals and it was the earliest method for treating waste PCBs in China. However, the pollution incidents in the towns of Guiyu and Taizhou had indicated it was not a suitable method. Abundant toxic wastes, such as heavy metals and persistent organic pollutants, were produced^18^. Meanwhile, the chemical reagents brought security risks to workers and ecosphere. Additionally, for leaching Au, hydrocyanic acid should be used, while it is one of the prohibited materials in industrial production^19^.

*Corona-electrostatic separation*: it was the preferred method for separating metals of waste PCBs. Less secondary pollution was generated. However, it was unsuitable to separate precious metals.

*Bioleaching:* bioleaching is an environment-friendly and low-cost method. However, the efficiency of bioleaching was rather low. Additionally, nonmetallic part of waste PCBs will destroy bioleaching process^14^.

**Figure 1:**
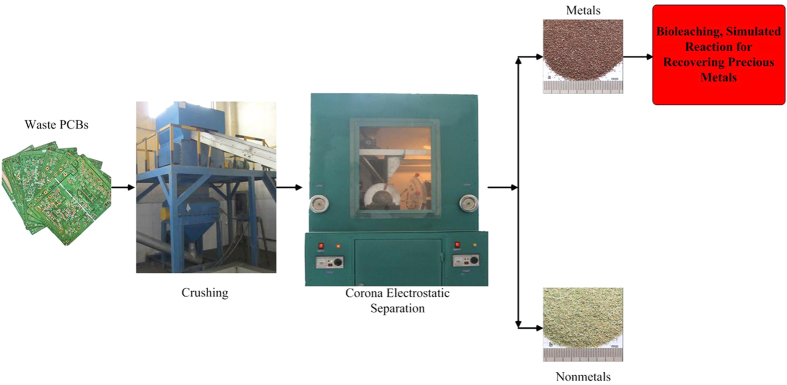
The flowchart of the hybrid technology for recovering precious metals from crushed waste PCBs.

Based on the above comparisons, a hybrid technology of physical and biological methods was proposed to separate precious metals from waste PCBs. The hybrid technology had three advantages. Firstly, corona-electrostatic separation separated nonmetals of crushed waste PCBs. It avoided the negative effect of nonmetals to bioleaching process. Secondly, bioleaching replaced chemical treatment to dissolve precious metals. It not only decreased production cost but also avoided security risks on workers and ecosphere. Thirdly, corona-electrostatic separation and bioleaching were both environment-friendly technologies. The flowchart of the hybrid technology was presented in Fig. 1. Waste PCBs were crushed into particles for liberating metals and nonmetals. Metallic and nonmetallic particles were separated by corona-electrostatic separation. Then, all the metallic particles were dissolved in bioleaching. At last, the metals were substituted out by the greater active metals from metallic lixivium in sequence.

In the hybrid technology, corona-electrostatic separation and substitution reaction were mature technologies in production. The key point of the hybrid technology was bioleaching. The urgent demands were to find the suitable microbe for dissolving metals and create bioreactor to improve the efficiency of bioleaching.

### Bioreactor Design

**Figure 2:**
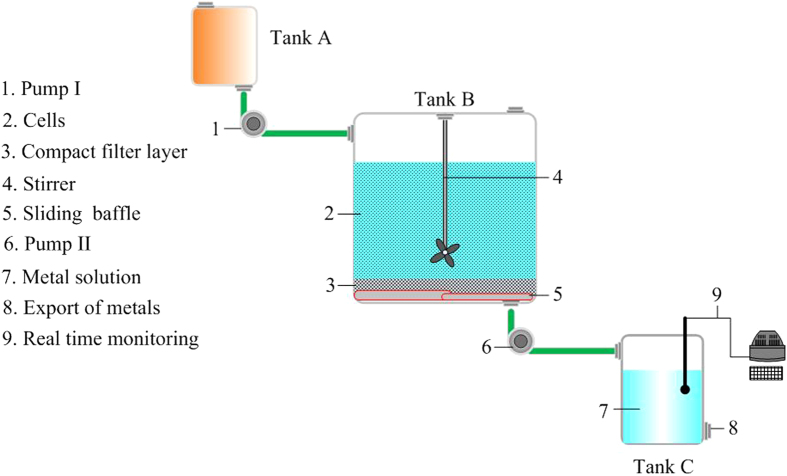
The structure chart of the designed bioreactor.

A novel bioreactor was designed to improve the efficiency of bioleaching for recovering precious metals from crushed waste PCBs. It was comprised of Tank A (volume 5 L), Tank B (volume 10 L), and Tank C (volume 5 L) (see Fig. 2). Tank A was designed to culture strain with nutrient solution. Volume of nutrient solution was set as 4 L. Cyanogenic strain was inoculated into Tank A in clean environment with no other microbes (operated in aseptic operation box). Culture time of cyanogenic strain in Tank A was set as 12 hours. The dissolved oxygen was provided by importing oxygen through aerator regularly. Then, Cyanogenic strain was pumped from Tank A into Tank B when it cultured 12 hours and produced the most mass of CN^−^. Mixed metallic particles from crushed waste PCBs were fed into Tank B. The mass ratio of the mixed metallic particles to the strain solution was 3–10: 1038. The composition of nutrient solution in Tank B were the same as that in Tank A. Sliding baffle was set up at the bottom of Tank B, and it was covered by a compact filter layer. Bioleaching process of mixed metals would begin in Tank B. Active metal was dissolved first. Inert metal would not be dissolved until active metal was dissolved completely. For obtaining high dissolution rate of metals, the bioleaching time (retention time) of the strain in Tank B should be controlled. Because the strain produced abundant CN^−^ in a limited amount of time. Thus, after a limited bioleaching time, the sliding baffle was opened, and the lixivium of active metal was pumped from Tank B to Tank C under the filtering of the compact filter layer. The inert metal and parts of active metal were remained in tank B. Additionally, improving the flow of pump II in Fig. 2 could avoid the blocking of the filter layer due to the microbial cells and its extracellular polymers. Meanwhile, periodic cleaning of the filter layer could keep the leachate removed to Tank C successfully. The above process was repeated for separating active metal until the real time monitoring system detected inert metal in Tank C. When inert metal ion was found in Tank C, it meant that the active metal had been separated completely. Then, inert metal solution began to be flowed into Tank C after active metal solution was pumped out from Tank C. At last, active metal and inert metal solutions were collected respectively. Active metal and inert metal could be obtained from their solutions by replacement reaction of other activity metals.

## Materials and Methods

### The employed strain

Cyanogenic strain was employed in bioleaching process of dissolving precious metals (such as Au and Ag) in waste PCBs. Precious metals were dissolved in to solution by CN^−^ produced by cyanogenic strains. Chemical equation of the dissolution process was presented as:


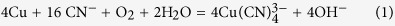



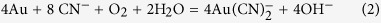


*Chromobacterium violaceum* (*C. violaceum*) could release CN^−^ in its growth and it could be used for dissolving Au^16^. However, *C. violaceum* is a disease-causing microbe, which is not suitable to be employed in industrial production.

**Figure 3:**
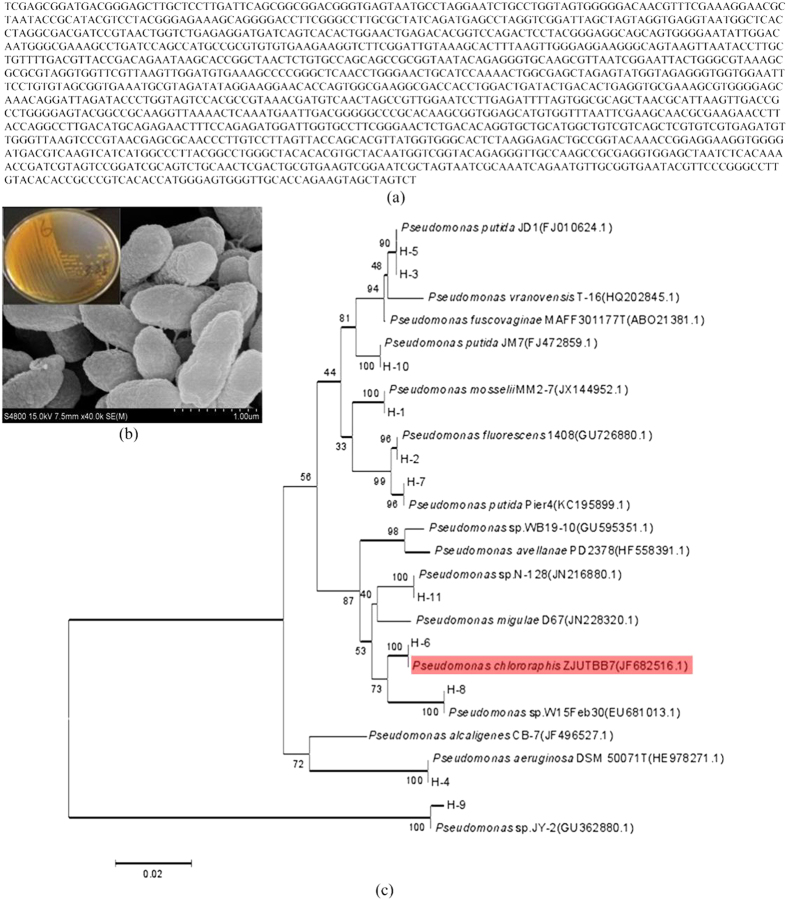
(a) Gene sequence of the employed strain (*P. chlororaphis*) for the bioleaching of precious metals in waste PCBs; (b) SEM of *P. chlororaphis*; (c) Phylogenetic tree of *P. chlororaphis* by using 16S rRNA gene sequences.

A new strain, *Pseudomonas chlororaphis* (*P. chlororaphis*) presented in Fig. 3, was found for dissolving precious metals from crushed waste PCBs in previous work^20^. It was not a disease-causing microbe and was suitable for being employed in industrial production. *P. chlororaphis* was enriched using soils obtained from the mining regions. 0.2 ml soil extract (1 g soil dissolved in 10 ml sterile water) was inoculated to NB culture medium (comprised of beef extract, peptone, NaCl, and agar) which containing sterile penicillin, novobiocin, and cycloheximide. After being cultured 72 hours, various strains of *Pseudomonas* were selected and purified. Color reaction was adopted to identify which strains can produce CN^−^. The strain having the ability of producing CN^−^, would make the yellow test paper (dipped in the solution of 0.5% picric acid and 2% sodium carbonate) turn to red. Turning time and color grade showed the ability of producing CN^−^. The DNAs of *Pseudomonas strains* were extracted by freeze-thaw method^20^. Then the DNAs were amplified using the following primers of 27F(5′-AGAGTTTGATCCTGGCTCAG-3′) and 1492R(5′-GGTTACCTTGTTACGACTT-3′), and the amplicons were sequenced. The conditions for amplification were: 94 °C pre-denaturation 5 min; 94 °C denaturation 30 s; 65 °C annealing 30 s; 72 °C amplification 90 s; 30 cycles; 72 °C amplification 10 min. According to the gene sequences, homology analyses of the strains were performed by comparing to GenBank data using software DNAMAN. Then, phylogenetic tree of the strains were constructed by software MEGA 5. *P. chlororaphis* was identified as the strain having the highest ability of producing CN^−^.

**Figure 4:**
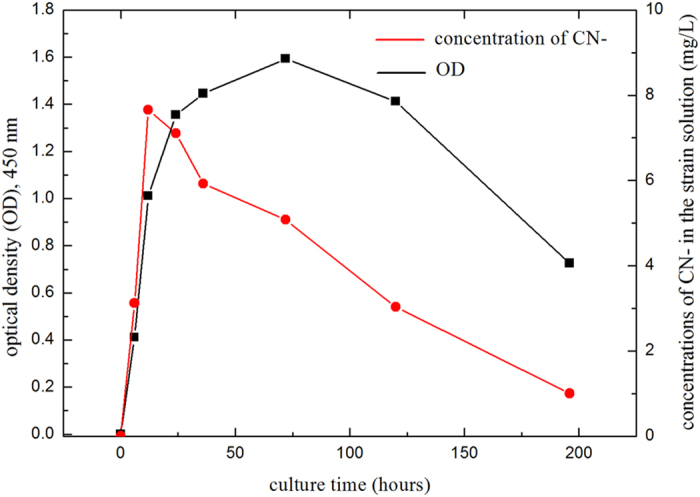
Flowchart of bioleaching experiments simulated to bioreactor operation for dissolving mixed particles of Cu and Au.

The highest concentration of CN^−^ released from *P. chlororaphis* could reach 7.66 mg/L, which was close to the highest concentration (7.8 mg/L) produced by *C. violaceum*^21^. The concentration of CN^−^ released from *P. chlororaphis* and its optical density (OD) are shown in Fig. 4. When the strain was cultured 12 hours, the value of OD was 1.011 and concentration of CN^−^ in nutrient reached the highest level of 7.66 mg/L. When the strain was cultured 72 hours, the value of OD reached the highest level of 1.593, the concentration of CN^−^ decreased to 5.08 mg/L. Then, when cultured 196 hours, the value of OD decreased to 0.752 and the concentration of CN^−^ drop to 0.98 mg/L. Culture medium for *P. chlororaphis* was comprised of NB medium, glycine, and methionine. NB medium was comprised of beef extract (0.5 wt.%), peptone (1 wt.%), sodium chloride (0.5 wt.%); distilled water (96 wt.%). Additionally, *P. chlororaphis* had a special nature that concentration of the produced CN^−^ became more and more lower, even down to zero, after the later stage of logarithmic growth phase. . The reason was not found. However, this phenomenon offered a way to treat the residual CN^−^ when all the metals were leached out. If the residual CN^−^ was not treated, it might harm workers and environment. Residual CN^−^ could be consumed by *P. chlororaphis*. It was a big advantage than chemical leaching and *C. violaceum* on environmental performance.

### Bioleaching of mixed Cu and Au particles

Bioleaching experiments of mixed Cu and Au particles were performed in flask. Particles of Au and Cu were fed into the flask. The samples of Cu and Au were pure metals, provided by reagent supplier, and metallic purities were 99.98% and 99.97% respectively. For simulating the particle size of crushed waste PCBs, the size of Cu and Au particles was set up as 0.6 mm. Then, the *P. chlororaphis* was inoculated into the flask. The experiment conditions were pH 7, temperature 25 °C, adding glycine (4.4 g/L) +methionine (2 g/L), and rotation speed of 60 r/min. The conditions of temperature and rotation speed were provided by temperature-control incubation table. These conditions could bring the greatest ability of producing CN^−^. Concentration of Au^+^ in solution was detected by the method of Inductively Coupled Plasma Optical Emission Spectrometer (ICP-OES). Concentrations of Cu^2+^ in solution were tested by the method of atomic absorption pectrometry (AAS)^22^.

### Orthogonal experiments of bioleaching simulated to bioreactor operation for separating Cu from Au particles

Orthogonal experimental design is an efficient experimental method for studying the influence of different factors in different levels^6^. We used this method to study influencing factors of *P. chlororaphis* for dissolving metals in bioleaching process. In previous work, influencing factors of *P. chlororaphis* for producing CN^−^ were pH, temperature, additive, and rotation speed. The optimized conditions producing highest concentration of CN^−^ were pH 7, temperature 25 °C, adding glycine (4.4 g/L) +methionine (2 g/L), rotation speed of 60 r/min. One of the purposes of rotation was to maintain the level of dissolved oxygen in the solution. The other was to stir the solution for better dissolution rate. Therefore, the factors of pH, temperature, and rotation speed were chosen as the influencing factors of *P. chlororaphis* for dissolving metals. In order to investigate the variation trend of metal dissolution rates caused by different levels of these three factors, four levels of the factors were chosen. Table L_16_(4^3^) was employed in the orthogonal experiments of bioleaching. Levels of the factors were given in Table 1. These four levels were chosen based on optimized conditions of *P. chlororaphis* for bringing the greatest ability of producing CN^−^. Bioleaching experiments were performed according to design of table L_16_(4^3^).

**Figure 5:**
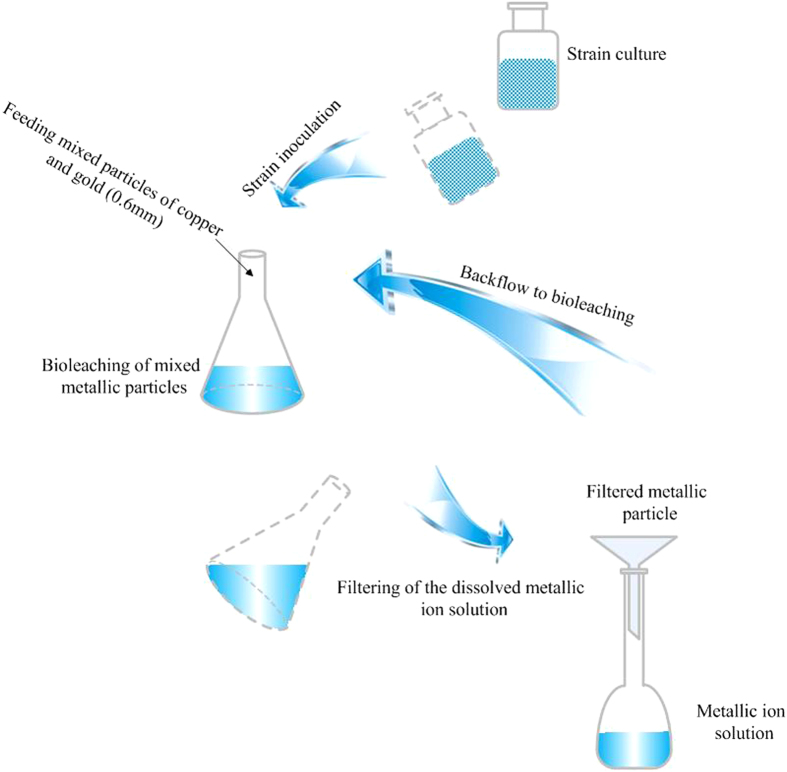
Bioleaching results of mixed metals of Cu and Au.

The process of bioleaching experiment was given in Fig. 5. This process simulated to the operation of the designed bioreactor. Particles (size 0.6 mm) of Au (0.3 g) and Cu (90 g) were fed into the flask. The mass proportion between Au (0.3 g) and Cu (90 g) was 1:300 which agreed with the proportion in waste PCBs of mobile phone. Then, after culturing 12 hours (when the strain had the greatest ability of producing CN^−^), *P. chlororaphis* was inoculated into the flask. Cu and Au particles began to be dissolved by CN^−^ secreted from *P. chlororaphis*. The solution in flask was filtered in to volumetric flask every 3 hour, and concentrations of Cu^2+^ and Au^+^ in the filtered solution were investigated. The aim of removing metallic ion solution by filtering was to improve the leaching rate. Then, the filtered metallic particles were back flowed to bioleaching flask. Concentrations of Au^+^ and Cu^2+^ in metallic ion solution were detected. Previous work had indicated that *P. chlororaphis* could dissolve both Cu and Au into solution. However, chemical activity of Cu was far greater than Au, so CN^−^ chosen to dissolve Cu firstly. Meanwhile, because of having greater activity, Cu substituted the dissolved Au from Au-hydrocyanic compound. Thus, when Au^+^ was detected in the filtered metallic ion solution, it meant that no Cu particles were remained. In other words, Cu particles had been dissolved out completely. Therefore, bioleaching time of Cu was considered as the leaching rate of separating Cu from Au particles in the orthogonal experiments of simulation bioleaching.

## Results and Discussion

### Bioleaching results of Au and Cu

**Figure 6:**
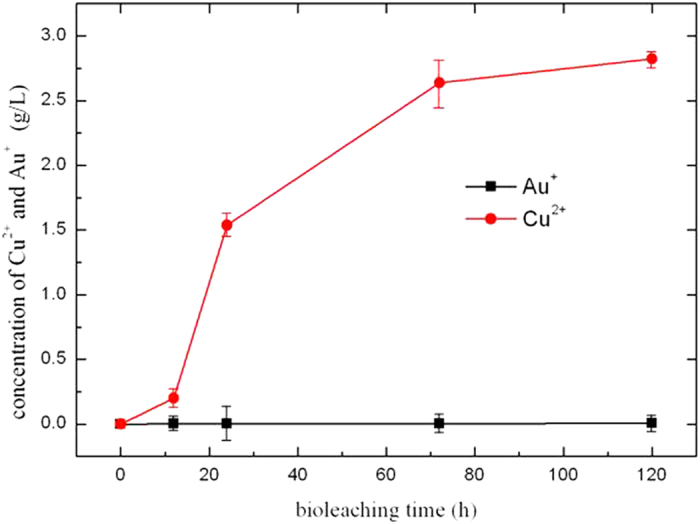
Tendency charts of the levels of every factor in orthogonal experiments of bioleaching.

Bioleaching results of mixed particles of Au and Cu were presented as Fig. 6. The results showed concentration of Au^+^ in nutrient solution was less than 0.006 mg/L. This concentration almost could be neglected. On the other hand, concentration of Cu^2+^ in nutrient solution reached 2823 mg/L at the culture time of 120 h, and still increased as the increasing of culture time. It indicated dissolution rate of Au could be neglected when Cu was existed. This phenomenon provided the feasibility to separate Cu from Au.

### Results of orthogonal experiments of bioleaching simulated to bioreactor operation and the recovery rates of Cu and Au

**Figure 7:**
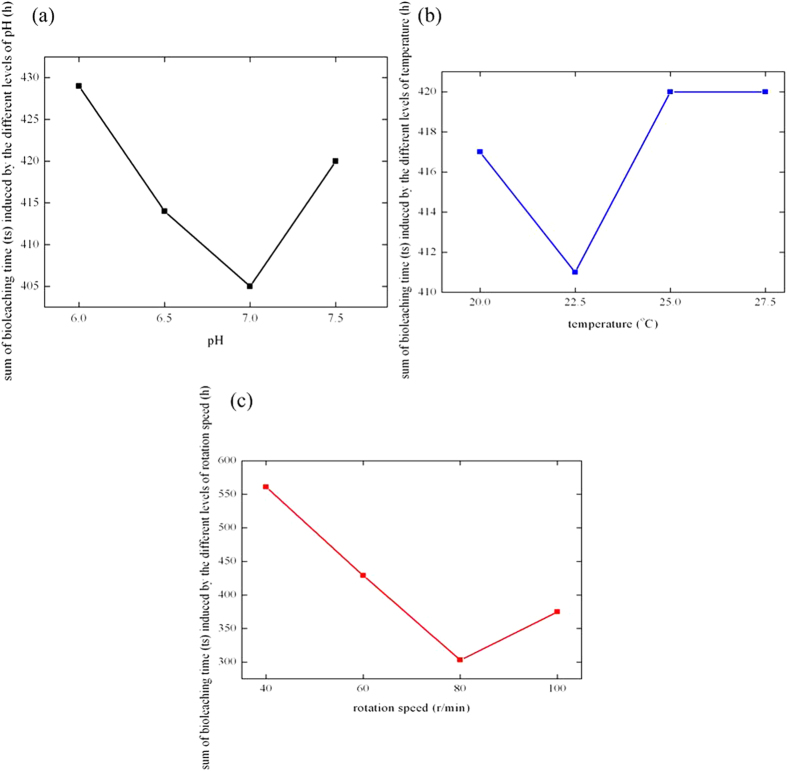
Tendency charts of the levels of every influencing factor in orthogonal experiments of bioleaching.

Results of orthogonal experiments were presented in Table 2. Bioleaching time of Cu was the investigation target of orthogonal experiments. Shorter bioleaching time of Cu meant the higher leaching rate for separating Cu from Au. Meanwhile, ranges between different sums of bioleaching time of Cu (*ts*) influenced by different levels of every factor were also given in Table 2. Greater value of range indicated the factor was more important to influence the bioleaching time of Cu. Therefore, *ω* was the critical factor, pH was general factor, and temperature was subordinate factor. The influencing sequence of the factors was *ω* > pH > temperature. Furthermore, we defined the levels of every factor as abscissa; set the sum of bioleaching time caused by every factor in different levels as ordinate; and then tendency charts of the levels of every factor were given in Figure 7. Seen from Figure 7a, bioleaching time decreased as the pH increased. When pH was 7.0, bioleaching time of Cu had the minimum value. When pH was greater than 7.0, the bioleaching time became greater. It indicated the optimized pH for *P. chlororaphis* to bioleach Cu was 7.0. Figure 7b indicated that bioleaching time decreased as the increasing of temperature. When temperature was 22.5 °C, bioleaching time of Cu had the minimum value. When temperature was greater than 22.5 °C, the bioleaching time became greater. It indicated that the optimized temperature for *P. chlororaphis* to bioleach Cu was 22.5 °C. Seen from Figure 7c, bioleaching time decreased as the increasing of rotation speed. When rotation speed was 80 r/min, bioleaching time of Cu had the minimum value. When rotation speed was greater than 80 r/min, the bioleaching time became greater. It indicated that the optimized rotation speed for *P. chlororaphis* to bioleach Cu was 80 r/min. Additionally, when rotation speed increased to 100 r/min, bioleaching time was prolonged. It showed although increasing of rotation speed could improve the level of dissolve oxygen in solution, too high speed would inhibit the bioleaching of metals by *P. chlororaphis.*

Based on the optimized parameters of pH 7.0, temperature 22.5 °C, additives of glycine (4.4 g/L) and methionine (2 g/L), and rotation speed 80 r/min, bioleaching experiments for recovering mixed Cu and Au particles were performed. Particles of Au (0.3 g) and Cu (90 g) were fed into the flask. Then, *P. chlororaphis* was inoculated into the flask after culturing 12 hours. Cu was firstly dissolved by CN^−^ produced by *P. chlororaphis*. The solution in flask was filtered in to volumetric flask every 3 hours, and concentrations of Cu^2+^ in the filtered solution were investigated. Then, the filtered metallic particles were back flowed to bioleaching flask. About 92.9 L Cu^2+^ solutions were obtained and concentration of Cu^2+^ in the solution was 0.8532 g/L. When Au^+^ was detected in the filtered solution, it meant that Cu particles had been dissolved completely. Then, under the repeating bioleaching of Au, Au^+^ solution was collected. About 153.8 L Au^+^ solutions were obtained and the concentration of Au^+^ was 0.00149 g/L. Recovery rates of Au and Cu were investigated. The results indicated that 79.31 g Cu and 0.23 g Au were obtained from the mixed Cu (90 g) and Au (0.3 g) particles. Recovery rates of Cu and Au reached 88.1% wt and 76.6% wt, respectively. The residual Cu and Au were lost such as adsorbed by the cell of *P. chlororaphis* in bioleaching process.

## Conclusion

For separating precious metal and improving the additional value of recovering waste PCBs, a novel hybrid technology of corona-electrostatic separation and bioleaching was proposed. A new bioreactor was designed for the bioleaching process. *P. chlororaphis*, which had strong ability of producing CN^−^, was proposed to be used for bioleaching precious metals from crushed waste PCBs. Bioleaching experiments for recovering mixed Cu and Au particles were performed. 88.1% wt Cu and 76.6% wt Au were recovered. This paper provided novel bioreactor and experimental data for guiding the industrial production of recovering precious metals from waste PCBs.

## Additional Information

**How to cite this article**: Jujun, R. *et al.* A Novel Designed Bioreactor for Recovering Precious Metals from Waste Printed Circuit Boards. *Sci. Rep.* **5**, 13481; doi: 10.1038/srep13481 (2015).

## Figures and Tables

**Table 1:** Levels of the factors in orthogonal experiment.

levels	factors		
	pH	Temperature (°C)	*ω* (r/min)
1	6.0	20.0	40
2	6.5	22.5	60
3	7.0	25.0	80
4	7.5	27.5	100

**Table 2:** Range analysis of the results of bioleaching experiments that followed the design of L_16_(4^3^).

experiments	pH	Temperature (°C)	*ω*(r/min)	*t*(h)
1	6.0	22.5	80	78
2	7.0	27.5	40	138
3	6.5	27.5	80	75
4	7.5	22.5	40	138
5	6.0	25.0	40	144
6	7.0	20.0	80	72
7	6.5	20.0	40	141
8	7.5	25.0	80	78
9	6.0	20.0	100	96
10	7.0	25.0	60	105
11	6.5	25.0	100	93
12	7.5	20.0	60	108
13	6.0	27.5	60	111
14	7.0	22.5	100	90
15	6.5	22.5	60	105
16	7.5	27.5	100	96
Sum of *ts* influenced by level 1 (h)	429	417	561	Total
Sum of *ts* influenced by level 2 (h)	414	411	429	
Sum of *ts* influenced by level 3 (h)	405	420	303	
Sum of *ts* influenced by level 4 (h)	420	420	375	
range	24	9	258	1668
